# Effect of PBO–FRCM Reinforcement on Stiffness of Eccentrically Compressed Reinforced Concrete Columns

**DOI:** 10.3390/ma13051221

**Published:** 2020-03-09

**Authors:** Tomasz Trapko, Michał Musiał

**Affiliations:** Faculty of Civil Engineering, Wroclaw University of Science and Technology, Pl. Grunwaldzki 11, 50-377 Wroclaw, Poland; michal.musial@pwr.edu.pl

**Keywords:** column, stiffness, FRCM, PBO mesh, PBO–FRCM

## Abstract

This paper examines the effect of PBO (P-phenylene benzobisoxazole)–FRCM (Fabric Reinforced Cementitious Matrix) reinforcement on the stiffness of eccentrically compressed reinforced concrete columns. Reinforcement with FRCM consists of bonding composite meshes to the concrete substrate by means of mineral mortar. Longitudinal and/or transverse reinforcements made of PBO (P-phenylene benzobisoxazole) mesh were applied to the analyzed column specimens. When assessing the stiffness of the columns, the focus was on the effect of the composite reinforcement itself, the value and eccentricity of the longitudinal force and the decrease in the modulus of elasticity of the concrete with increasing stress intensity in the latter. Dependences between the change in the elasticity modulus of the concrete and the change in the stiffness of the tested specimens were examined. The relevant standards, providing methods of calculating the stiffness of composite columns, were used in the analysis. For columns, which were strengthened only transversely with PBO mesh, reinforcement increases their load capacity, and at the same time, the stiffness of the columns increases due to the confinement of the cross-section. The stiffness depends on the destruction of the concrete core inside its composite jacket. In the case of columns with transverse and longitudinal reinforcement, the presence of longitudinal reinforcement reduces longitudinal deformations. The columns failed at higher stiffness values in the whole range of the eccentricities.

## 1. Introduction

The FRCM (Fabric Reinforced Cementitious Matrix) system, in which a composite mesh is bonded to the substrate with mineral mortar, is becoming the preferred method of choice in increasing the load capacity of concrete elements or the method in repairing them. FRCM reinforcements are applied to strengthen or repair concrete and reinforced concrete elements subjected to bending, shearing and compression. Increasingly more experimental and theoretical investigations into the behavior of elements strengthened with a composite mesh on mineral mortar are reported [1,2,3,4,5,6,7,8].

The FRCM system is characterized by higher resistance to elevated temperatures than the FRP (Fiber Reinforced Polymer) system in which non-metallic composite fibers are embedded in epoxy resin [9,10]. An additional advantage of the FRCM system is its better compatibility with the concrete substrate in comparison with FRP systems, where the composite tends to separate from the substrate. FRCM reinforced structural elements show more plastic behavior than the ones strengthened with FRP. This is attributed to the slip, which occurs in the mortar–fiber interface. In the FRCM system’s behavior, one can distinguish two main phases separated by the moment at which the cement mortar cracks. Sometimes an intermediate phase, connected with the initial development of cracks in the matrix, can be distinguished. The behavior of the composite in its prior-to-cracking state depends on both the fibers and the matrix. The behavior of this reinforcement in its post-mortar fail state depends mainly on the fibers [11,12,13,14].

The knowledge about the considered subject can be extended through tests and analyses of the confined concrete elements subjected to compression [15,16,17,18,19,20,21,22,23,24,25,26]. The effectiveness of the composite reinforcement is determined by its stiffness, which affects the ratio of transverse (circumferential) strains to longitudinal strains. The stiffness of the compressed columns determines its ability to deform plastically and redistribute the internal forces in the structure. The concrete core’s compressibility is limited, and when the limit is exceeded, the ability of the core to resist the increasing load diminishes, which initiates its failure. As long as longitudinal stresses *σ*_c_ in the concrete do not exceed its compressive strength *f*′_co_, the strains in the composite remain low. From the instant when *σ*_c_ > *f*′_co_, the transverse stresses in the composite increase, so does the PBO mesh action on the concrete core. In order to develop a general method of dimensioning columns reinforced with FRCM, it is necessary to determine the effect of this reinforcement on the change in the stiffness of such columns with increasing stresses.

Tests [17,18,19,20] (on which the present analyses are based) carried out by the authors on reinforced concrete columns reinforced with PBO mesh on mineral mortar (PBO–FRCM) show that the reinforced columns are characterized by greater ductility than unreinforced columns. Longitudinal FRCM reinforcement improves the ductility of eccentrically compressed columns. The presence of longitudinal composite reinforcement brings about an increase in the longitudinal stiffness of the columns and consequently affects the ultimate compressive strain value. In reinforced concrete columns longitudinally and transversely reinforced with PBO–FRCM, the ultimate strain value increases with increasing eccentricity and depends on the number of transverse reinforcement layers.

Ombres and Verre [23] carried out tests on reinforced concrete columns reinforced with PBO mesh on mineral mortar. They analyzed the effectiveness of the PBO–FRCM reinforcement in increasing the load capacity of the tested elements, focusing on the effect of eccentric loading and transverse reinforcement intensity on the structural response of the confined (wrapped) columns. In the first series of columns, the load was applied eccentrically to the top of the specimens, whereas the reaction force at their base was eccentrically applied on the other side of the longitudinal axis of the columns. In the second test series, the eccentric load was applied to the top and base of the specimens on the same side of the longitudinal axis of the columns. It was found that the PBO–FRCM confinement (winding) increased the load capacity of the columns by 20–39% relative to the unconfined columns. For comparison, in experimental studies on reinforced concrete columns with only a transverse winding (C_1H and C_2H) carried out by the present authors [18,19], a 5–24% increase in load capacity, where the load capacity values were dependent on the number of reinforcement layers and the eccentricity value, was obtained. Ombres and Verre [23] also showed that the increase in compressive strains is linear until the peak load is reached. This finding is important for the present authors since it corroborates the research results presented [19] and provides the basis for the current analyses of the change in the stiffness of columns reinforced with PBO–FRCM.

### 1.1. Flexural Stiffness of Compressed Reinforced Concrete Columns

Research into the behavior of compressed columns shows that the stiffness of their cross-sections is not constant. When evaluating the stiffness of such elements, one should take into account the effect of the longitudinal force and its eccentricity and obviously the decrease in the modulus of elasticity of the concrete (*E*_c_) with the increasing load. The value of the modulus of elasticity of the concrete (*E*_c_) in a given stress state is highest at a stress close to zero. As the stress increases, the modulus of elasticity of the concrete decreases. For pure concrete at longitudinal stresses *σ*_c_ > 0.5*f*′_co_, the ratio of the concrete’s instantaneous modulus of elasticity *E*_c,time_ to its initial modulus of elasticity *E*_0_ amounts to about 0.77 [27]. At higher stresses, this ratio is difficult to estimate since the concrete enters the plastic phase characteristic related to its grade.

### 1.2. Standard Analysis of Stiffness of Compressed Reinforced Concrete Columns

Bearing in mind the similarity of PBO–FRCM reinforced columns to composite steel–concrete columns made of steel tubes filled with concrete (CFST—Concrete Filled Steel Tube), let us recall the most important standards providing methods of calculating the stiffness of CFST columns.

According to Eurocode 4 [28], the value of characteristic effective flexural stiffness (*EI*)_eff_ of the cross-section of a composite column should be calculated from the formula:

(1) $${\left(EI\right)}_{eff}=E_{a}I_{a}+E_{s}I_{s}+K_{e}E_{cm}I_{c},$$

where *K*_e_ is a (correction) factor reducing the stiffness component originating from the cross-section of the concrete, amounting to 0.6 according to the standard. Eurocode 4 does not directly specify what this correction factor includes, but certainly it does not include long-term effects. The latter are taken into account through the reduction of the modulus of elasticity of the concrete from *E*_cm_ to *E*_c,eff_.

Eurocode 2 [29], recommends to calculate the stiffness of slender columns with any cross-section from the formula:

(2) $$EI=K_{c}E_{cd}I_{c}+K_{s}E_{s}I_{s},$$

where *K*_c_ is a coefficient dependent on the effects of cracking and creep. Moreover, the Eurocode 2 [29], recommends to use *E*_cd,eff_ instead of *E*_cd_ for statically indeterminate columns.

(3) $$E_{cd,eff}=\frac{E_{cd}}{\left(1+\varphi_{ef}\right)}$$

When, for the purposes of the analyses, one omits the effect of the creep of the concrete, *K*_c_ can be calculated from the relation, given in [29]:

(4) $$K_{c}=k_{1}k_{2},$$

where:

(5) $$k_{1}=\sqrt{\frac{f_{ck}}{20}},$$

(6) $$k_{2}=\left(\frac{P}{A_{c}f_{cd}}\right)\left(\frac{\lambda }{170}\right)\leq 0.2.$$

As it is apparent, this coefficient takes into account the strength parameters of the concrete, the slenderness of the column and most importantly, as applied in this paper, the stress intensity of the member, whereas it does not take into account the effect of the eccentric load.

*K*_s_ is a factor for contribution of steel reinforcement—*K*_s_ = 1, 0 when *ρ* ≥ 0.002 and *K*_s_ = 0 when *ρ* ≥ 0.01.

## 2. Test Specimens

**Figure 1:**
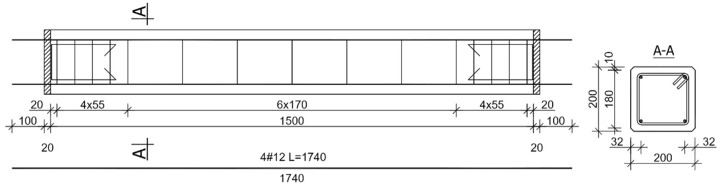
Test specimens.

In order to determine the effect of the PBO–FRCM reinforcement on the change in the stiffness of columns strengthened in this way, 1500 (height) × 200 × 200 column specimens were subjected to tests, the results of which were presented in more detail in [18,19] (Figure 1). The spacing of the stirrups was concentrated, at the element ends to 1/3 of the spacing, over a section longer than 200 mm (the cross-section size of the column). In order to ensure the parallelism of the holding-down planes and uniform pressure on concrete and reinforcement bars in the columns, front metal plates were made through. The longitudinal concrete steel reinforcement was made of four ∅12 bars (RB500W, *f*_yk_ = 500 MPa) [29] and the transverse concrete steel reinforcement had the form of ∅6 stirrups (St0S, *f*_yk_ = 220 MPa) [29]. All of the columns were prefabricated. All of the test columns and concrete specimens were made from a single concrete batch during mixing and vibrating at the concrete prefabrication plant. The concrete-mix design is shown in Table 1.

The specimens were made of concrete with mean cubic compressive strength *f*_cm,cube_ = 55.5 MPa, mean cylinder compressive strength *f*_cm,cyl_ = 48.7 MPa and mean modulus of elasticity *E*_cm_ = 33.8 GPa. The mechanical properties of the concrete were determined by standard tests [30,31].

Ruredil X Mesh Gold PBO (P-phenylene benzobisoxazole) mesh (Ruredil, San Donato Milanese, Italy) and mineral mortar Ruredil X Mesh M750 (Ruredil, San Donato Milanese, Italy) were used as the composite reinforcement [32,33,34]. The mesh is a two-way woven sheet on a matrix, in which there are four times more fibers in the primary direction than in the perpendicular direction. The specifications of the PBO–FRCM strengthening materials are given in Table 2.

Column specimens simultaneously strengthened longitudinally with one layer of the mesh and transversely with one (1H) or two (2H) layers of the mesh were selected for the investigations. The particular layers of this reinforcement were made of a single continuous PBO mesh sheet. The longitudinal composite reinforcement was laid with its fibers running parallel to the column’s axis. The columns were wrapped such that the fibers ran horizontally in the primary direction. In the specimens of type C_1V1H and C_1V2H, first, the longitudinal layer was made and then the horizontal layers were laid. The successive layers of mesh were separated from one another with layers of mortar. The length of the finish mesh overlap amounted to 100 mm and the overlap was located on the side perpendicular to the compression plane. The layers of the composite embedded in the binder were topped with a mortar layer closing and leveling the outer surface.

**Figure 2:**
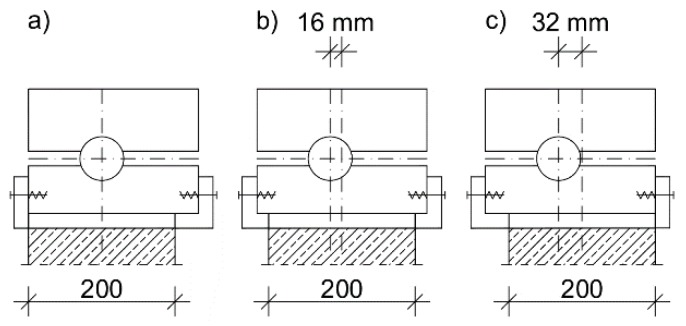
Cylindrical bearing used in the experiment: (**a**) e = 0; (**b**) e = 16 mm; (**c**) e = 32 mm.

The tests were carried out at the axial force eccentricity within the core of the cross-section amounting to 0, *h*/12 (16 mm) and *h*/6 (32 mm) (Figure 2). The distance between the cylinder axes (rotational axes of the columns) was 1690 mm. For each of the eccentricities, one of the columns was tested as the reference specimen without the C_C reinforcement (Table 3).

## 3. Experimental Results and Analysis

### 3.1. Changes in Elasticity Modulus of Concrete in Tested Columns

**Figure 3:**
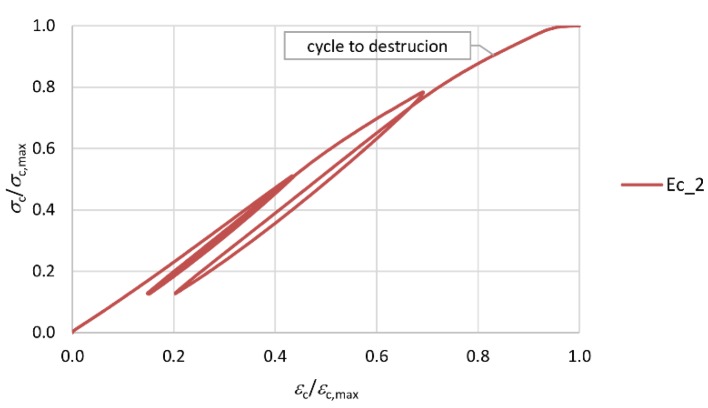
Method of determining modulus of elasticity of concrete.

The mean elasticity modulus *E*_cm_ = 33.8 GPa of the concrete of the columns was determined on five 350 mm cylindrical specimens with a diameter of 113 [31]. After the reference failure load had been determined, six initial load cycles up to the level of 0.5*σ*_c,max_, followed by one load cycle up to 0.8*σ*_c,max_ and the final cycle until failure, were carried out (Figure 3).

**Figure 4:**
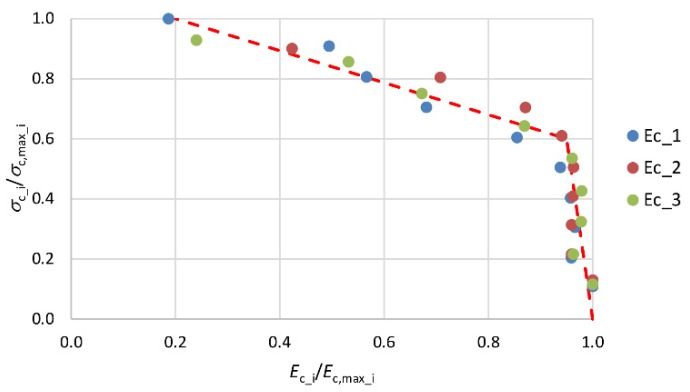
Character of change in secant elasticity modulus values as stresses in concrete increase (i—number of sample; i = 1, i = 2, i = 3).

From the ultimate load cycle dependence, *σ*_c_-*ε*_c_ secant elasticity modulus values were determined at every 0.1*σ*_c_ for the three selected specimens. Figure 4 shows how the elastic modulus values change as the stresses in the concrete increase. The relative elasticity modulus *E*_c_i_/*E*_c,max_i_ and stress *σ*_c_i_/*σ*_c,max_i_ values in the concrete were determined by relating them to the maximum value for a given specimen (i = 1, 2 and 3).

Figure 4 shows that up to the stress level of about 0.6*f*_c,cyl_ the elasticity modulus values increase only slightly (by about 5%). At higher stress values of σ_c_ > 0.6*f*_c,cyl_, the elasticity modulus values decrease more sharply until the minimum value of about 0.2*E*_c,max_ is reached immediately before failure. The character of this change can be linearly described, as shown by the broken line in Figure 4.

### 3.2. Change in Stiffness of Columns with PBO–FRCM Reinforcement

**Figure 5:**
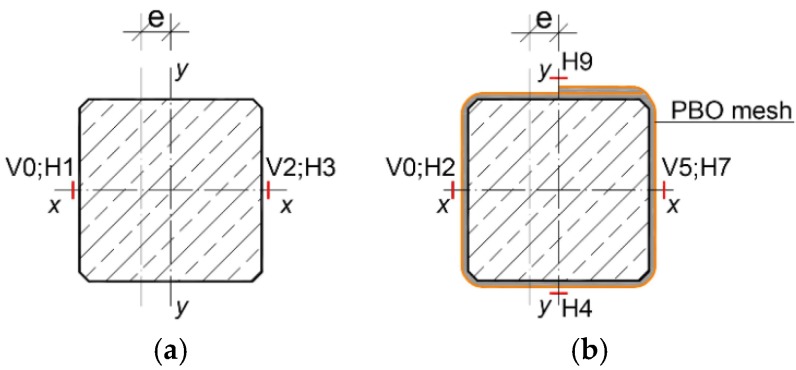
Instrumentation for columns. (**a**) reference columns; (**b**) reinforced columns.

As the columns were being tested, the longitudinal and transverse strains were measured by strain gauges arranged along the circumference of the columns at half of their height. Depending on the column type, different arrangements of strain gauges were adopted. In the reference columns C_C_0, C_C_16 and C_C_32, two vertical strain gauges, V0 and V2, and two horizontal strain gauges, H1 and H3, were used. Strain gauges V0 and H1 were located on the side where the force acted at the eccentricity (the more compressed side) (Figure 5a). Six strain gauges were used in the case of columns C_1H, C_2H, C_1V1H and C_1V2H. Two vertical strain gauges, V0 and V5, were located in the plane of compression. The next four strain gauges, H2, H4, H7 and H9, measured circumferential strains (Figure 5b).

**Figure 6:**
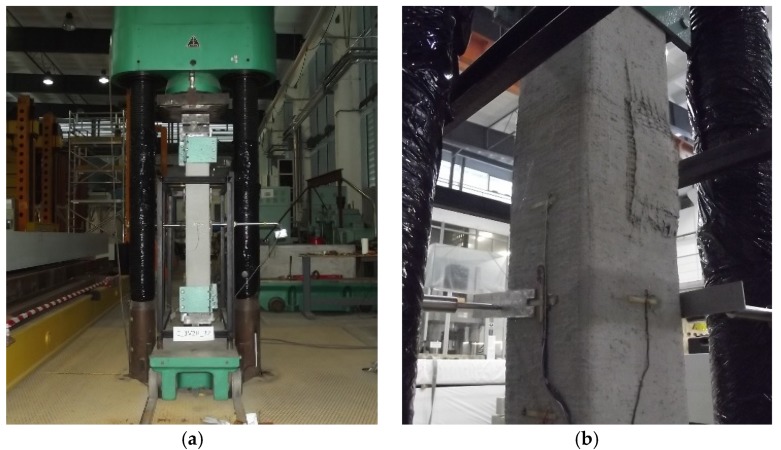
C_1V2H_32 specimen on test stand. (**a**) test setup; (**b**) failure of column

The horizontal columns’ displacements (deflections) were measured by Linear Variable Differential Transformers (LVDTs, HBM Masstechnik, Darmstadt, Germany). The measurement span of the transducers was ±10 mm. The LVDTs were mounted on a separate steel frame while the measurement took place at half the height of the columns (Figure 6) [19]. The columns were tested until failure under monotonically increasing displacement. The load, strains and horizontal displacements were acquired with an automatic data acquisition system.

The curvatures of the specimens were calculated from Equation (7) on the basis of the measured maximum longitudinal strains *ε*_v2,lim_ and *ε*_v1,lim_ on the more and less compressed (tensioned) sides of the cross-section, respectively.

(7) $$\frac{1}{r}=\frac{\varepsilon_{v2,lim}-\varepsilon_{v1,lim}}{h},$$

While analyzing the value of longitudinal strains *ε*_v_, in the confined columns with the PBO mesh only, with horizontal layout fibers over the main direction (C_1H and C_2H), failure was observed at a comparable level of strain (Table 4). For the columns in the group C_1H, the limit compression strains amount to 2.736‰–2.962‰; the values in group C_2H amount to 2.827‰–3.200‰. In both columns groups that were loaded at the core limit, C_1H_32 and C_2H_32, at the failure stage, there occurred tension on the side opposite to the action of load. The presence of the longitudinal strengthening reduces the limit strains *ε*_v2_ of axially compressed columns at which point the destruction of the section occurs, which is fairly unfavorable. For instance, in the element C_1H_0, the strain *ε*_v2,max_ = 2.736‰, and the additional longitudinal strengthening in the element C_1V1H_0, resulted in a decrease in these strains to *ε*_v2,max_ = 2.392‰. In contrast, the strains for the elements C_2H_0 and C_1V2H_0 were recorded: *ε*_v2,max_ = 3.200‰ and *ε*_v2,max_ = 1.734‰, respectively. The impact of the longitudinal PBO mesh on the limit values of compression strains is evident in the element groups C_1V1H and C_1V2H. It is evident in both groups C_1V1H and C_1V2H that eccentrically compressed elements are capable of transferring considerably higher compression strains on the side of the action of force than axially compressed elements. In addition, the value of these strains rises jointly with the rise in eccentricity.

**Figure 7:**
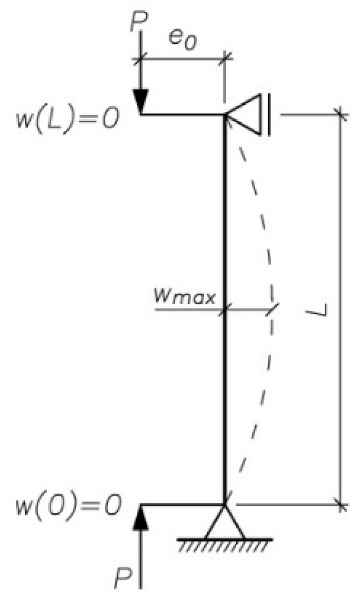
Static diagram of column.

The bending moments at the instant of failure (*M*_max_) were calculated from (8) on the basis of the maximum deflections *w*_max_ (Figure 7).

(8) $$M_{max}=P_{max}\cdot \left(e_{0}+w_{max}\right)$$

The bending stiffness of columns can be numerically analyzed with the use of Bernoulli’s hypothesis with or without eccentric load and additional reinforcements such as fiber materials. The additional longitudinal composite reinforcements contribute to the increasing bending stiffness directly and transverse composite reinforcements give confinement effect to increase stiffness. The axial stiffness should not be evaluated under the combination of axial force and bending moment.

Assuming that Bernoulli’s hypothesis is applicable in this case (plane section remains plane) and starting with the general dependence between the curvature of the specimen’s deformed axis (1/*r*), bending moment *M*_max_ and bending stiffness *EI* (9), the change in stiffness was analyzed depending on the type of strengthening of the column and the stress intensity in the latter.

(9) $$\frac{1}{r}=\frac{M_{max}}{EI}.$$

**Figure 8:**
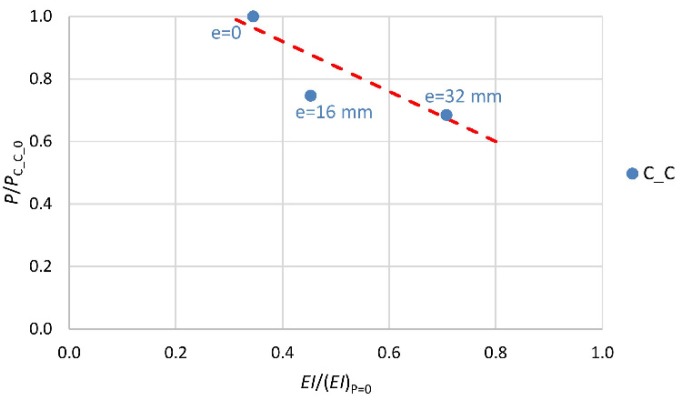
Change in stiffness of reference specimens depending on intensity of their stress.

**Figure 9:**
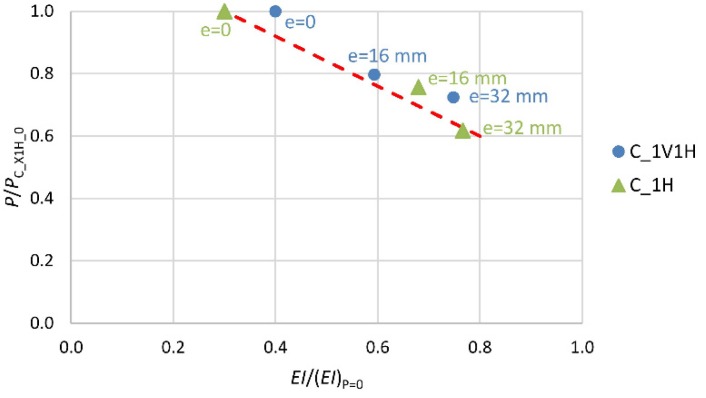
Change in stiffness of specimens with single layer of transverse composite reinforcement (X—number of layers of longitudinal composite reinforcement V: 0 or 1).

**Figure 10:**
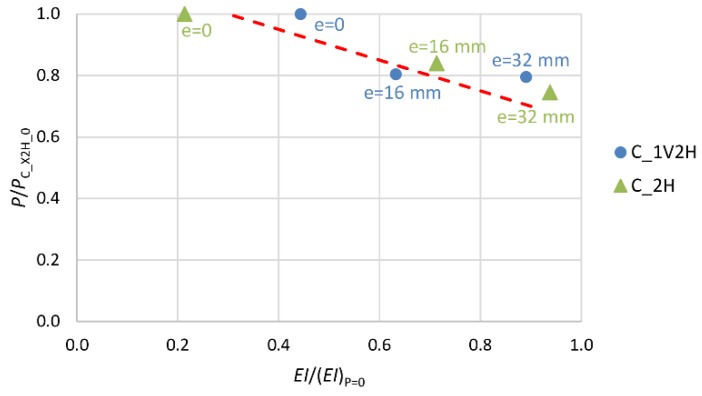
Change in stiffness of specimens with two layers of transverse composite reinforcement (X—number of layers of longitudinal composite reinforcement V: 0 or 1).

The next three diagrams (Figure 8, Figure 9 and Figure 10) show the change (decrease) in the stiffness of the analyzed columns depending on their stress intensity. The horizontal axis represents the ratio of column stiffness at failure *EI* to initial column stiffness (*EI*)_P=0_ for the load eccentricity of, respectively, 0, 16 and 32 mm. The vertical axis represents the ratio of the ultimate force to the load capacity of the axially compressed column in a given group for the load eccentricity of 0, 16 and 32 mm. The broken line marks the trend in stiffness change.

In the case of column C_C_0 (most stressed), the stiffness at the point of failure amounts to 35% of the initial value (Figure 8). For the unstrengthened columns loaded at the initial eccentricity of 16 mm and 32 mm (C_C_16 and C_C_32), which were put under less stress, the stiffness at the point of failure amounts to, respectively, 45% and 71% of the initial stiffness value. The elasticity modulus value of the “plain concrete” decreases until about 0.2*E*_c,max_ before failure. The smaller decrease in stiffness of the reinforced concrete columns, than that resulting from the change in the elasticity modulus of the “plain concrete” itself, is evident due to the presence of the longitudinal reinforcement and the shape of the cross-section of the columns.

A similar trend in the change of stiffness is observed in the columns with a single layer (1H) of transverse composite reinforcement (Figure 9). The addition of another layer (2H) of transverse composite reinforcement results in greater stiffness of the composite jacket, and so of the whole cross-section (Figure 10). This is illustrated by the slope of the trend line in the two diagrams.

The stiffness of the composite jacket in these investigations is defined with the equivalent modulus of elasticity of the PBO–FRCM strengthening according to the following formula:

(10) $$E_{1}=\frac{t}{R}\cdot E_{f},$$

where *E*_f_ is given in Table 2 and *R* is the radius of a circle with the circumference equals the circumference of a considering cross-section. For the considered columns with the square cross-section with the side length *a*:

(11) $$R=\frac{4\cdot a}{2\cdot \pi }.$$

One should note here that in comparison to the reference columns, the decreases in load capacity were observed for columns C_1V2H_0 and C_1V2H_16 (Table 5) [18]. This is not surprising as it was caused by the increase in the stiffness of the columns due to the little-deformable composite jacket. The longitudinal composite reinforcement reduces the longitudinal deformability of the columns, which is rather disadvantageous. Stiffer transverse composite reinforcement reduces the ability of the columns to deform (deflect) in the bending plane. This observation applies particularly to axially compressed columns at a slight eccentricity. An analysis of the diagrams shows that the stiffness of the columns strengthened with PBO mesh on mineral mortar depends on the intensity of stress in the concrete confined by the composite jacket. The stress intensity depends on the eccentricity, which equals the sum of the initial eccentricity and the deflection of the column.

**Figure 11:**
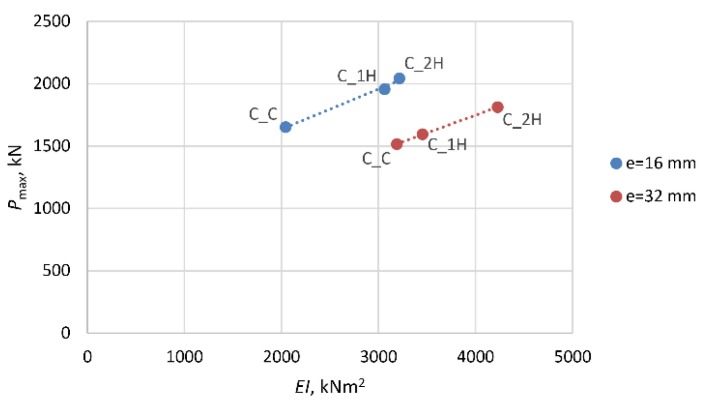
Change in stiffness of columns C_1H and C_2H as a function of failure load.

**Figure 12:**
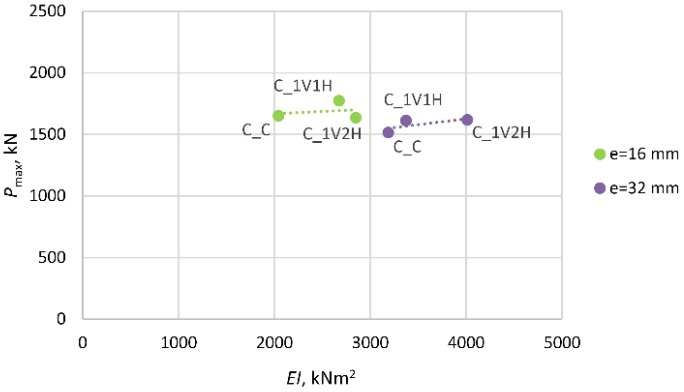
Change in stiffness of columns C_1V1H and C_1V2H as a function of failure load.

The next two diagrams (Figure 11 and Figure 12) and Table 5 show the change in the stiffness of the columns as a function of the maximum (ultimate) force registered in the course of the tests. In Figure 11, which illustrates the behavior of the columns strengthened only transversely, one can see that the introduction of one (1H) or two (2H) layers of transverse composite reinforcement results in an increase in the stiffness of the composite jacket. The stiffness of the columns in the state of the ultimate bearing capacity depends on the intensity of stress in the cross-section at the instance of failure. The stress intensity does not increase geometrically with the number of strengthening layers. The columns with longitudinal composite reinforcement behave completely differently (Figure 12). In this case, the columns’ stiffness is determined by the presence of the longitudinal composite reinforcement. The lower stress intensity, in comparison with the specimens of type C_1H and C_2H, is accompanied by a reduction in the flexural rigidity of the columns. The application of composite reinforcement along the axis of the columns resulted in an increase in their longitudinal stiffness. Both types of columns: C_1V1H and C_1V2H show considerably greater ductility than the corresponding columns without longitudinal composite reinforcement C_1H and C_2H. This is reflected in the lower value of stiffness at failure at lower stress intensities, in comparison with the columns of type C_1H and C_2H.

**Figure 13:**
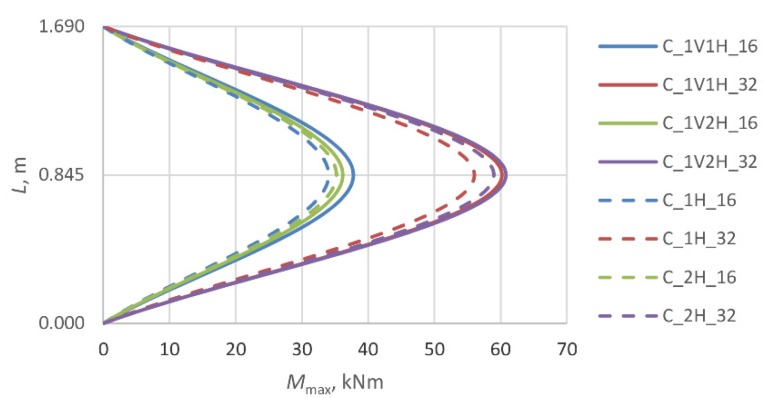
The bending moment of the eccentrically loaded columns.

The ductility of the columns in these investigations is defined as the ability to horizontally displace the columns, which is induced with the bending moments (eccentric load) what is presented in Figure 13. *M*_max_ is the first-order moment. The slenderness ratio of the RC columns *λ* < *λ*_lim_ according to [29].

**Figure 14:**
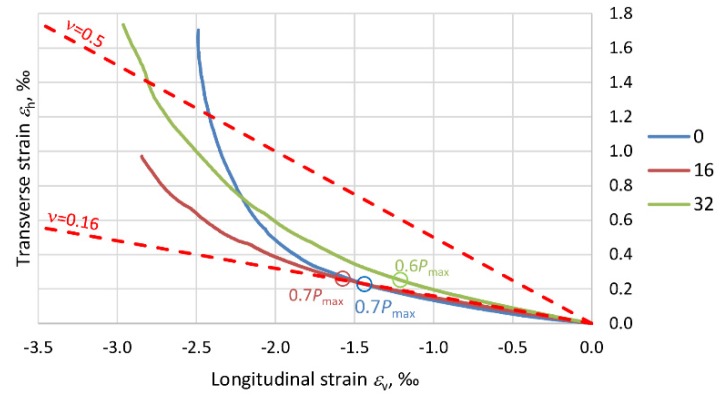
Graphs showing the variation of Poisson’s ratio *ν* for columns C_1H.

**Figure 15:**
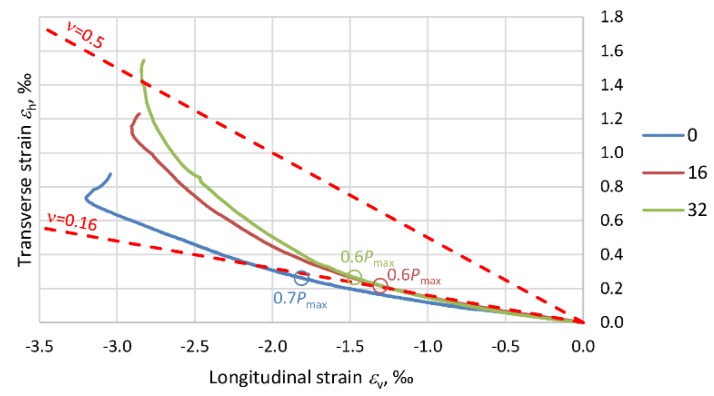
Graphs showing the variation of Poisson’s ratio *ν* for columns C_2H.

**Figure 16:**
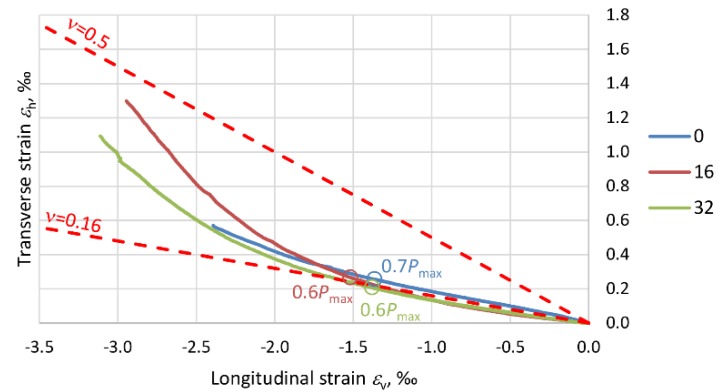
Graphs showing the variation of Poisson’s ratio *ν* for columns C_1V1H.

**Figure 17:**
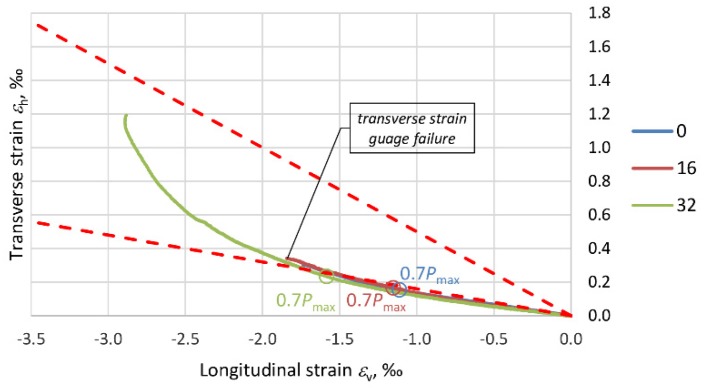
Graphs showing the variation of Poisson’s ratio *ν* for columns C_1V2H.

The effect of the composite jacket in PBO–FRCM columns is closely connected with the variation in the elasticity modulus of the concrete (*E*_c_) due to the stress destruction of the concrete core. Microcracks develop in the concrete beyond the level of stress in the column at which Poisson’s ratio *ν* is no longer a liner [35]. As a result of the damage, the load-carrying surface area is reduced and consequently the stiffness of the member decreases. The next graphs (Figure 14, Figure 15, Figure 16 and Figure 17) show Poisson’s ratio versus eccentricity for the analyzed columns. One can see that beyond a certain stress value, Poisson’s ratio *ν* quickly increases, which is due to the extensive microcracking of the concrete core. This stress level corresponds to 60–70% of the maximum (ultimate) force *P*_max_ observed during the tests.

As the stress further increases, the rate of volumetric changes begins to fall. The concrete is no longer a continuous body, undergoes disintegration and is held only by the external composite jacket. This situation lasts until the reinforcement at the end of the overlap of the PBO mesh starts to delaminate. In the columns strengthened only transversely, i.e., C_1H and C_2H, the Poisson ratio exceeds 0.5, and the volumetric strain assumes negative values. In the case of columns C_1V1H and C_1V2H, the effect of the longitudinal PBO mesh (reducing the compressive stress increment) is clearly visible and ratio *ν* < 0.5.

With regard to the variability of PBO–FRCM column stiffness, the variation in the ratio of transverse strain to longitudinal strain (Poisson’s ration *ν*), due to the destruction of the concrete inside the composite jacket should be taken into account in the standards.

## 4. Conclusions

The determination of the stiffness of columns strengthened with composite materials (PBO mesh on mineral mortar in the considered case) is a difficult and complicated task. One cannot adopt, a priori, the standard regulations dedicated to reinforced concrete or composite elements to determine the stiffness of such columns. The aim of the investigations presented in this paper was to assess the effect of the PBO–FRCM reinforcement of eccentrically compressed columns on their stiffness depending on the level of stress intensity.

In the case of unstrengthened columns, the expected results were measured. As eccentricity increased, the longitudinal ultimate force decreased and the deflection of the columns increased. As the curvature of the columns increased, so did the bending moment at half of their height. Each of the columns failed at a correspondingly lower intensity of the stress in the whole cross-section, which was produced by applying longitudinal force on the more compressed side.

In the case of columns C_1H and C_2H, which were strengthened only transversely, our measurements and evidence collected point to the fact that this kind of reinforcement increases their load capacity, but at the same time, the stiffness of the columns increases due to the confinement of the cross-section. In comparison with the reference (unstrengthened) specimens, the curvature would decrease at the initial eccentricities after the first and second PBO layers were laid. This was accompanied by an increase in the ultimate force and a decrease in the horizontal deflection of the columns. At eccentricities of 16 and 32 mm, the columns failed at correspondingly higher stiffness values than the reference members, owing to the use of transverse composite reinforcement. Whereas in the case of columns C_1H_0 and C_2H_0, their load capacity was reached at lower stiffness values, which indicates considerable destruction of the concrete core under axial compression.

In the considered case of quadrangular columns, the tri-axial state of stress induced by the confinement of the concrete is insignificant and the addition of another layer of transverse composite reinforcement only increases the stiffness of the composite jacket.

Unfortunately, in the case of columns C_1V1H and C_1V2H, their higher ductility and greater horizontal deflectability in the bending plane do not directly translate into an increase in their load capacity in comparison with the columns without longitudinal composite reinforcement. As shown in the earlier research, longitudinal PBO–FRCM reinforcement improves the ductility of eccentrically compressed columns. The presence of this reinforcement, however, reduces longitudinal deformations at which the mesh ruptures, which is disadvantageous. Thanks to the presence of longitudinal PBO reinforcement, the columns failed at higher stiffness values in the whole range of the eccentricities: 0, 16 and 32 mm.

This paper investigated the effect of PBO–FRCM reinforcement on the stiffness of eccentrically compressed RC columns. The dependencies between the change in the elasticity modulus of the concrete and the change in the stiffness of the tested specimens were examined. This subject is of great practical relevance. Few such experiments are available in the literature. The analyses presented in this paper can be used to design a wider series of tests of PBO–FRCM columns with various types of reinforcement, which are subjected to eccentric compression. The results of such tests can be used to formulate a standard relation for a column stiffness reduction coefficient depending on the type of reinforcement, the longitudinal force and the cross-section’s stress intensity.

## Figures and Tables

**Table 1:** Concrete mixture.

Portland cement of grade 42.5R	455 kg/m^3^
Sand 0–2 mm	710 kg/m^3^
Aggregate 2–8 mm (river pebble)	710 kg/m^3^
Aggregate 8–16 mm (river pebble)	610 kg/m^3^
Water	200 kg/m^3^
Superplasticizer	0.75% cement mass—3.375 kg/m^3^
*w*/*c* ratio	0.44

**Table 2:** Mechanical and geometrical characteristic of the PBO–FRCM strengthening materials.

Parameter	Unit	PBO Mesh [32,33]	Cement Based Matrix [32,33]	PBO–FRCM System [34]
Tensile strength	(MPa)	5800	-	1664
Compressive strength	(MPa)	-	29.0	-
Young modulus	(GPa)	270	6.0	128
Nominal thickness	(mm)	0.0455 longitudinal	-	-
		0.0224 transversal	-	-

**Table 3:** Configuration of specimens [18,19].

Specimens	Cross-Section	Height	Internal Reinforcement	FRCM Type		Eccentricity
				Horizontal 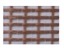	Vertical 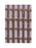	
	(mm)	(mm)	-	-	-	(mm)
C_C_0	200 × 200	1500	4∅12	No	No	0
C_C_16				No	No	16
C_C_32				No	No	32
C_1H_0				1 layer	No	0
C_1H_16				1 layer	No	16
C_1H_32				1 layer	No	32
C_2H_0				2 layers	No	0
C_2H_16				2 layers	No	16
C_2H_32				2 layers	No	32
C_1V1V_0				1 layer	1 layer	0
C_1V1V_16				1 layer	1 layer	16
C_1V1V_32				1 layer	1 layer	32
C_1V2H_0				2 layers	1 layer	0
C_1V2H_16				2 layers	1 layer	16
C_1V2H_32				2 layers	1 layer	32

**Table 4:** Summary of testing results.

Column	Vertical Strain at Peak Load	
	*ε*_v2,max_	*ε*_v2,max_
	(‰)	(‰)
C_C_0	−2.121	−1.752
C_C_16	−3.183	−0.052
C_C_32	−3.135	+0.369
C_1H_0	−2.736	−2.459
C_1H_16	−2.845	−0.622
C_1H_32	−2.962	+0.283
C_2H_0	−3.200	−1.489
C_2H_16	−2.907	−0.715
C_2H_32	−2.827	+0.106
C_1V1H_0	−2.392	−1.762
C_1V1H_16	−2.941	−0.115
C_1V1H_32	−3.112	+0.460
C_1V2H_0	−1.734	−1.510
C_1V2H_16	−1.842	−0.903
C_1V2H_32	−2.890	+0.477

**Table 5:** Change in stiffness of columns C_1H, C_2H, C_1V1H, C_1V2H as a function of failure load.

Specimens	*EI*	*P*_max_
	(kNm^2^)	(kN)
C_C_0	1556	2214
C_1V1H_0	1801	2227
C_1V2H_0	1999	2035
C_C_16	2042	1652
C_1V1H_16	2673	1775
C_1V2H_16	2850	1636
C_C_32	3188	1516
C_1V1H_32	3371	1613
C_1V2H_32	4013	1618
C_C_0	1556	2214
C_1H_0	1352	2587
C_2H_0	962	2434
C_C_16	2042	1652
C_1H_16	3062	1957
C_2H_16	3214	2044
C_C_32	3189	1516
C_1H_32	3454	1596
C_2H_32	4226	1812

## Nomenclature

*A*_c_	Concrete cross-section, m^2^
*E*_a_	Modulus of elasticity of structural steel, gpa
*E*_c_	Modulus of elasticity of concrete, gpa
*E*_cm_	Secant modulus of elasticity of concrete, gpa
*E*_cd_	Design value of modulus of elasticity of concrete, gpa
*E*_f_	Modulus of elasticity of PBO–FRCM strengthening system, gpa
*E*_s_	Modulus of elasticity of reinforcing steel, gpa
*I*_c_	Moment of inertia of concrete, cm^4^
*I*_s_	Moment of inertia of reinforcing steel, cm^4^
*I*_a_	Moment of inertia of structural steel, cm^4^
*P*	Load, kn
*e*_0_	Initial eccentricity, mm
*f*_c,cyl_	Cylindrical compressive strength of concrete, mpa
*f*_c,cube_	Cubic compressive strength of concrete, mpa
*f*′_co_	Compressive strength of unconfined concrete, mpa
*f*_ck_	Characteristic value of concrete compressive strength, mpa
*f*_cd_	Design value of concrete compressive strength, mpa
*f*_u_	Ultimate strength of PBO mesh, mpa
*f*_y_	Yield strength of steel, mpa
*f*_t_	Ultimate tensile strength of steel, mpa
*t*	Nominal thickness of PBO mesh, mm
*w*	Column deflection, mm
*λ*	Column slenderness,
*ε*_c_	Strain in concrete, ‰
*ρ*	Reinforcement ratio for longitudinal reinforcement
*σ*_c_	Stress in concrete, mpa
*ϕ*_ef_	Effective value of the creep coefficient

## References

[1] Ombres L. (2011). Flexural analysis of reinforced concrete beams strengthened with a cement based high strength composite material. Compos. Struct..

[2] Ombres L. (2011). The structural performances of PBO-FRCM strengthened RC beams. Struct. Build..

[3] Ombres L. Shear Capacity of Concrete Beams Sstrengthened with Cement Based Composite Mmaterials. Proceedings of the 6th International Conference on FRP Composites in Civil Engineering (CICE 2012).

[4] Loreto G., Babaeidaarabad S., Leardini L., Nanni A. (2015). RC beams shear-strengthened with fabric-reinforced-cementitious-matrix (FRCM) composite. Int. J. Adv. Struct. Eng..

[5] Trapko T., Urbańska D., Kamiński M. (2015). Shear strengthening of reinforced concrete beams with PBO-FRCM composites. Compos. Part B.

[6] Ombres L. (2015). Structural performances of reinforced concrete beams strengthened in shear with a cement based fibre composite material. Compos. Struct..

[7] Trapko T., Musiał M. (2017). PBO mesh mobilization via different ways of anchoring PBO-FRCM reinforcements. Compos. Part B.

[8] Nerilli F., Ferracuti B. On Tensile Behavior of FRCM Materials: An Overview. Proceedings of the 8th International Conference on Fibre-Reinforced Polymer (FRP) Composites in Civil Engineering (CICE 2016).

[9] Trapko T. (2010). The influence of temperature on the durability and effectiveness of strengthening of concrete with CFRP composites. Eng. Build..

[10] Trapko T. (2013). The effect of high temperature on the performance of CFRP and FRCM confined concrete elements. Compos. Part B.

[11] D’Antino T., Carloni C., Sneed L.H., Pellegrino C. (2014). Matrix-fibre bond behaviour in PBO FRCM composites: A fracture mechanics approach. Eng. Fract. Mech..

[12] D’Ambrisi A., Feo L., Focacci F. (2013). Experimental analysis on bond between PBO-FRCM strengthening materials and concrete. Compos. Part B.

[13] Carozzi F.G., Poggi C. (2015). Mechanical properties and debonding strength of Fabric Reinforced Cementitious Matrix (FRCM) systems for masonry strengthening. Compos. Part B.

[14] Ombres L. (2015). Analysis of the bond between Fabric Reinforced Cementitious Mortar (FRCM) strengthening systems and concrete. Compos. Part B.

[15] Ombres L. (2007). Confinement Effectiveness in Concrete Strengthened with Fibre Reinforced Cement Based Composite Jackets. Proceedings of the FRPRCS-8: 8th International Symposium on Fibre-Reinforced Polymer Reinforcement for Concrete Structures, Patras, Greece, 16–18 July 2007.

[16] De Caso y Basalo F.J., Matta F., Nanni A. (2012). Fibre reinforced cement-based composite system for concrete confinement. Constr. Build. Mater..

[17] Trapko T. (2013). Fibre Reinforced Cementitious Matrix confined concrete elements. Mater. Des..

[18] Trapko T. (2014). Behaviour of Fibre Reinforced Cementitious Matrix strengthened concrete columns under eccentric compression loading. Mater. Des..

[19] Trapko T. (2014). Effect of eccentric compression loading on the strains of FRCM confined concrete columns. Constr. Build. Mater..

[20] Trapko T. (2014). Confined concrete elements with PBO-FRCM composites. Constr. Build. Mater..

[21] Colajanni P., De Domenico F., Recupero A., Spinella N. (2014). Concrete columns confined with fibre reinforced cementitious mortars: Experimentation and modelling. Constr. Build. Mater..

[22] Colajanni P., Fossetti M., Macaluso G. (2014). Effects of confinement level, cross-section shape and corner radius on the cyclic behaviour of CFRCM confined concrete columns. Constr. Build. Mater..

[23] Ombres L., Verre S. (2015). Structural behaviour of fabric reinforced cementitious matrix (FRCM) strengthened concrete columns under eccentric loading. Compos. Part B.

[24] Ombres L. (2017). Concrete confinement with a cement based high strength composite material. Compos. Struct..

[25] Ombres L. (2017). Structural performances of thermally conditioned PBO FRCM confined concrete cylinders. Compos. Struct..

[26] Donnini J., Spagnuolo S., Corinaldesi V. (2019). A comparison between the use of FRP, FRCM and HPM for concrete confinement. Compos. Part B.

[27] Hoła J. (2000). Relation of Initiating and Critical Stress to Stress Failure. Scientific Papers of the Institute of Building Engineering of the Wroclaw University of Technology.

[28] Polish Standard PN-EN 1994-1-1:2008 (2008). Eurocode 4: Design of Composite Steel and Concrete Structures—Part 1-1: General Rules and Rules for Buildings.

[29] Polish Standard PN-EN 1992-1-1:2008 (2008). Eurocode 2. Design of Concrete Structures—Part 1-1: General Rules and Rules for Buildings.

[30] Polish Standard PN-EN 12390-3:2009 (2009). Testing Hardened Concrete. Part 3: Compressive Strength of Test Specimens.

[31] (2009). Testing Hardened Concrete. Part 13: Determination of Secant Modulus of Elasticity in Compression.

[32] Ruredil X. (2009). Mesh Gold Data Sheet.

[33] (2004). Technical Approval.

[34] ACI Committee 549 (2013). Guide to Design and Construction of Externally Bonded Fabric-Reinforced Cementitious Matrix (FRCM) Systems for Repair and Strengthening Concrete and Masonry Structures.

[35] Neville A.M. (2012). Properties of Concrete.

